# Crystal structure of (*S*)-5,7-diphenyl-4,7-di­hydro­tetra­zolo[1,5-*a*]pyrimidine

**DOI:** 10.1107/S2056989015002996

**Published:** 2015-03-04

**Authors:** Ivy K. Price, Celine Rougeot, Jason E. Hein

**Affiliations:** a Chemistry and Chemical Biology, University of California, Merced, 5200 North Lake Road, Merced, CA 95343, USA

**Keywords:** crystal structure, tetra­zolo[1,5-*a*]pyrimidine, π–π stacking, hydrogen bonding

## Abstract

The title compound, C_16_H_13_N_5_, was synthesized by coupling amino­tetra­zole with chalcone in the presence of an amine organocatalyst derived from chincona alkaloid. There are two mol­ecules, *A* and *B*, in the asymmetric unit. In mol­ecule *A*, the dihedral angles between the partly hydrogenated pyrimidine ring system (r.m.s. deviation = 0.056 Å) and the *sp* ^2^- and *sp* ^3^-bonded phenyl groups are 33.32 (11) and 86.53 (11)°, respectively. The equivalent data for mol­ecule *B* are 0.049 Å, and 27.05 (10) and 85.27 (11)°, respectively. In the crystal, *A*+*B* dimers linked by pairs of N—H⋯N hydrogen bonds generate *R*_2_^2^(8) loops. The dimers are linked by aromatic π–π stacking inter­actions [shortest centroid–centroid separation = 3.5367 (15) Å], which results in a three-dimensional network.

## Related literature   

For background to tetra­zoles, see: Desenko *et al.* (2001 ▸); Ghorbani-Vaghei *et al.* (2013 ▸).
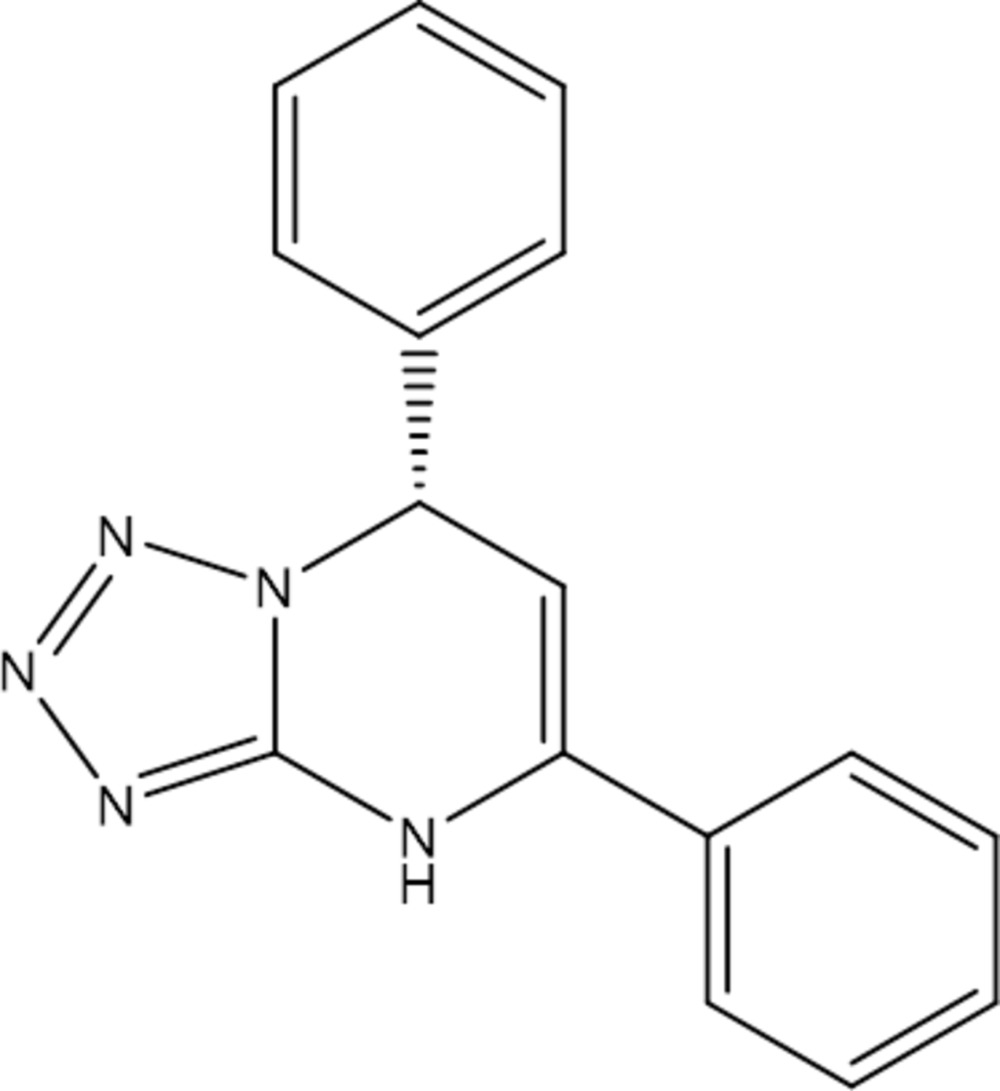


## Experimental   

### Crystal data  

C_16_H_13_N_5_
*M* *_r_* = 275.31
Monoclinic, 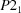

*a* = 8.7736 (2) Å
*b* = 8.8396 (2) Å
*c* = 17.6810 (4) Å
β = 98.8220 (9)°
*V* = 1355.03 (5) Å^3^
*Z* = 4
Cu *K*α radiation
μ = 0.68 mm^−1^
*T* = 100 K
0.35 × 0.20 × 0.14 mm

### Data collection  

Bruker D8 APEX Cu diffractometer
Absorption correction: multi-scan (*SADABS*; Bruker, 2013 ▸) *T*_min_ = 0.108, *T*_max_ = 0.818
21873 measured reflections
4926 independent reflections
4822 reflections with *I* > 2σ(*I*)
*R*_int_ = 0.035

### Refinement  

*R*[*F* ^2^ > 2σ(*F* ^2^)] = 0.054
*wR*(*F* ^2^) = 0.121
*S* = 1.08
4926 reflections
387 parameters
1 restraint
H atoms treated by a mixture of independent and constrained refinement
Δρ_max_ = 0.37 e Å^−3^
Δρ_min_ = −0.16 e Å^−3^
Absolute structure: Flack *x* determined using 2194 quotients [(*I* ^+^)−(*I* ^−^)]/[(*I* ^+^)+(*I* ^−^)] (Parsons & Flack, 2004 ▸)
Absolute structure parameter: 0.04 (13)

### 

Data collection: *APEX2* (Bruker, 2013 ▸); cell refinement: *SAINT* (Bruker, 2013 ▸); data reduction: *SAINT*; program(s) used to solve structure: *SHELXS97* (Sheldrick, 2008 ▸); program(s) used to refine structure: *SHELXL2013* (Sheldrick, 2015 ▸); molecular graphics: *OLEX2* (Dolomanov *et al.*, 2009 ▸); software used to prepare material for publication: *OLEX2*.

## Supplementary Material

Crystal structure: contains datablock(s) I. DOI: 10.1107/S2056989015002996/hb7347sup1.cif

Structure factors: contains datablock(s) I. DOI: 10.1107/S2056989015002996/hb7347Isup2.hkl

Supporting information file. DOI: 10.1107/S2056989015002996/hb7347Isup3.cdx

Supporting information file. DOI: 10.1107/S2056989015002996/hb7347Isup4.cml

**Fig. 1.**
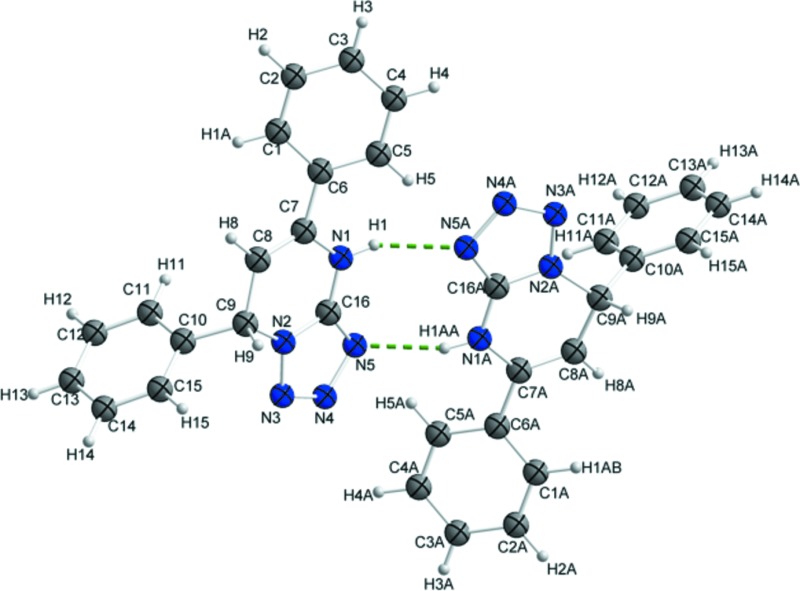
Asymmetric unit of (S)-5,7-diphenyl-4,7-dihydrotetrazol[1,5-a]pyrimidine, in ellipsoid thermal representation (50% of probability). The two molecule of the asymmetric unit are linked by hydrogen bonds (dashed green lines).

S . DOI: 10.1107/S2056989015002996/hb7347fig1.tif
Asymmetric unit of (*S*)-5,7-diphenyl-4,7-di­hydro­tetra­zol[1,5-a]pyrimidine, in ellipsoid thermal representation (50% of probability). The two mol­ecule of the asymmetric unit are linked by hydrogen bonds (dashed green lines).

CCDC reference: 1048927

Additional supporting information:  crystallographic information; 3D view; checkCIF report

## Figures and Tables

**Table 1:** Hydrogen-bond geometry (, )

*D*H*A*	*D*H	H*A*	*D* *A*	*D*H*A*
N1H1N5*A*	0.84(4)	2.16(4)	2.952(3)	157(3)
N1*A*H1*AA*N5	0.84(4)	2.10(4)	2.912(3)	163(4)

## supplementary crystallographic information

### S1. Experimental

(E)-Chalcone (0.4 g, 1.921 mmol) was dissolved in aceto­nitrile (9.6 ml) and heated to 80°C. 1H-tetra­zol-5-amine (0.163 g, 1.921 mmol) and di­phenyl hydrogen phosphate (0.096 g, 0.384 mmol) were then added as powders and stirred until the sample was homogenous. (R)-((1S,2R,4S,5R)-5-ethyl­quinuclidin-2-yl)(6-meth­oxy­quinolin-4-yl)methanamine, (0.063 g, 0.192 mmol) was then added and the reaction stirred at 80°C for 24h. The same was cooled to room temp and allowed to stand uncapped to permit slow evaporation. Crystals initially formed were isolated and recrystallized from di­chloro­methane as colourless blocks.

### S1.1. Refinement

Crystal data, data collection and structure refinement details are summarized in Table 1.

### Crystal data

**Table:**

C_16_H_13_N_5_	*F*(000) = 576
*M**_r_* = 275.31	*D*_x_ = 1.350 Mg m^−^^3^
Monoclinic, *P*2_1_	Cu *K*α radiation, λ = 1.54178 Å
*a* = 8.7736 (2) Å	Cell parameters from 9874 reflections
*b* = 8.8396 (2) Å	θ = 2.5–68.2°
*c* = 17.6810 (4) Å	µ = 0.68 mm^−^^1^
β = 98.8220 (9)°	*T* = 100 K
*V* = 1355.03 (5) Å^3^	Block, clear colorless
*Z* = 4	0.35 × 0.20 × 0.14 mm

### Data collection

**Table:**

Bruker D8 APEX Cu diffractometer	4926 independent reflections
Radiation source: Micro Focus Rotating Anode, Bruker FR-591	4822 reflections with *I* > 2σ(*I*)
Multilayer Mirrors monochromator	*R*_int_ = 0.035
Detector resolution: 8.0 pixels mm^-1^	θ_max_ = 68.2°, θ_min_ = 2.5°
φ and ω scans	*h* = −10→10
Absorption correction: multi-scan (*SADABS*; Bruker, 2013)	*k* = −10→10
*T*_min_ = 0.108, *T*_max_ = 0.818	*l* = −21→21
21873 measured reflections	

### Refinement

**Table:**

Refinement on *F*^2^	Secondary atom site location: difference Fourier map
Least-squares matrix: full	Hydrogen site location: mixed
*R*[*F*^2^ > 2σ(*F*^2^)] = 0.054	H atoms treated by a mixture of independent and constrained refinement
*wR*(*F*^2^) = 0.121	*w* = 1/[σ^2^(*F*_o_^2^) + (0.099*P*)^2^ + 0.0265*P*] where *P* = (*F*_o_^2^ + 2*F*_c_^2^)/3
*S* = 1.08	(Δ/σ)_max_ < 0.001
4926 reflections	Δρ_max_ = 0.37 e Å^−^^3^
387 parameters	Δρ_min_ = −0.16 e Å^−^^3^
1 restraint	Absolute structure: Flack *x* determined using 2194 quotients [(*I*^+^)-(*I*^-^)]/[(*I*^+^)+(*I*^-^)] (Parsons & Flack, 2004)
Primary atom site location: structure-invariant direct methods	Absolute structure parameter: 0.04 (13)

### Special details

**Table:**

Experimental. Absorption correction: SADABS-2012/1 (Bruker, 2013) was used for absorption correction. wR2(int) was 0.0856 before and 0.0465 after correction. The Ratio of minimum to maximum transmission is 0.1320. The λ/2 correction factor is 0.0015.
Geometry. All e.s.d.'s (except the e.s.d. in the dihedral angle between two l.s. planes) are estimated using the full covariance matrix. The cell e.s.d.'s are taken into account individually in the estimation of e.s.d.'s in distances, angles and torsion angles; correlations between e.s.d.'s in cell parameters are only used when they are defined by crystal symmetry. An approximate (isotropic) treatment of cell e.s.d.'s is used for estimating e.s.d.'s involving l.s. planes.

### Fractional atomic coordinates and isotropic or equivalent isotropic displacement parameters (Å^2^)

**Table:**

	*x*	*y*	*z*	*U*_iso_*/*U*_eq_	
N1	0.1241 (2)	0.7357 (3)	0.20818 (12)	0.0437 (4)	
H1	0.128 (4)	0.690 (4)	0.250 (2)	0.054 (9)*	
N2	0.1544 (2)	0.7090 (2)	0.07940 (12)	0.0433 (4)	
N3	0.2314 (2)	0.6164 (3)	0.03696 (12)	0.0467 (5)	
N4	0.3068 (3)	0.5225 (3)	0.08421 (12)	0.0476 (5)	
N5	0.2824 (2)	0.5492 (3)	0.15778 (12)	0.0458 (5)	
C1	−0.0730 (3)	1.0885 (3)	0.24985 (15)	0.0468 (5)	
H1A	−0.0506	1.1491	0.2085	0.056*	
C2	−0.1410 (3)	1.1537 (3)	0.30742 (15)	0.0499 (6)	
H2	−0.1652	1.2585	0.3052	0.060*	
C3	−0.1741 (3)	1.0677 (4)	0.36812 (15)	0.0508 (6)	
H3	−0.2212	1.1131	0.4074	0.061*	
C4	−0.1382 (3)	0.9151 (4)	0.37142 (15)	0.0530 (6)	
H4	−0.1598	0.8557	0.4133	0.064*	
C5	−0.0705 (3)	0.8486 (3)	0.31339 (15)	0.0489 (6)	
H5	−0.0469	0.7436	0.3157	0.059*	
C6	−0.0369 (3)	0.9346 (3)	0.25178 (14)	0.0432 (5)	
C7	0.0289 (3)	0.8631 (3)	0.18799 (14)	0.0424 (5)	
C8	−0.0012 (3)	0.9102 (3)	0.11511 (14)	0.0431 (5)	
H8	−0.0540	1.0037	0.1054	0.052*	
C9	0.0427 (3)	0.8252 (3)	0.04719 (13)	0.0423 (5)	
H9	−0.0515	0.7721	0.0211	0.051*	
C10	0.1090 (3)	0.9162 (3)	−0.01272 (14)	0.0416 (5)	
C11	0.2279 (3)	1.0198 (3)	0.00668 (13)	0.0438 (5)	
H11	0.2646	1.0408	0.0590	0.053*	
C12	0.2929 (3)	1.0926 (3)	−0.05011 (15)	0.0472 (5)	
H12	0.3754	1.1619	−0.0367	0.057*	
C13	0.2374 (3)	1.0641 (3)	−0.12674 (15)	0.0476 (5)	
H13	0.2808	1.1151	−0.1656	0.057*	
C14	0.1189 (3)	0.9613 (3)	−0.14640 (14)	0.0465 (5)	
H14	0.0813	0.9415	−0.1988	0.056*	
C15	0.0552 (3)	0.8874 (3)	−0.08959 (14)	0.0436 (5)	
H15	−0.0258	0.8165	−0.1032	0.052*	
C16	0.1852 (3)	0.6651 (3)	0.15244 (13)	0.0418 (5)	
N1A	0.2796 (3)	0.3097 (3)	0.27062 (12)	0.0457 (5)	
H1AA	0.287 (4)	0.364 (5)	0.232 (2)	0.054 (9)*	
N2A	0.1962 (2)	0.2931 (3)	0.39031 (12)	0.0440 (5)	
N3A	0.1134 (3)	0.3784 (3)	0.43317 (12)	0.0479 (5)	
N4A	0.0776 (3)	0.5003 (3)	0.39495 (12)	0.0477 (5)	
N5A	0.1334 (2)	0.4997 (3)	0.32690 (11)	0.0448 (5)	
C1A	0.4542 (3)	−0.0403 (3)	0.21227 (15)	0.0455 (5)	
H1AB	0.4120	−0.1095	0.2446	0.055*	
C2A	0.5287 (3)	−0.0939 (3)	0.15462 (15)	0.0480 (5)	
H2A	0.5370	−0.1999	0.1474	0.058*	
C3A	0.5921 (3)	0.0054 (3)	0.10666 (14)	0.0463 (5)	
H3A	0.6431	−0.0322	0.0669	0.056*	
C4A	0.5795 (3)	0.1605 (3)	0.11792 (14)	0.0465 (5)	
H4A	0.6229	0.2292	0.0858	0.056*	
C5A	0.5040 (3)	0.2156 (3)	0.17564 (14)	0.0441 (5)	
H5A	0.4956	0.3217	0.1826	0.053*	
C6A	0.4401 (3)	0.1156 (3)	0.22367 (13)	0.0436 (5)	
C7A	0.3557 (3)	0.1710 (3)	0.28455 (13)	0.0433 (5)	
C8A	0.3471 (3)	0.0932 (3)	0.34915 (14)	0.0449 (5)	
H8A	0.4061	0.0030	0.3576	0.054*	
C9A	0.2507 (3)	0.1381 (3)	0.40930 (13)	0.0443 (5)	
H9A	0.1593	0.0693	0.4054	0.053*	
C10A	0.3383 (3)	0.1305 (3)	0.49058 (13)	0.0446 (5)	
C11A	0.4812 (3)	0.1996 (3)	0.50895 (15)	0.0494 (6)	
H11A	0.5249	0.2526	0.4708	0.059*	
C12A	0.5608 (3)	0.1912 (3)	0.58352 (17)	0.0545 (6)	
H12A	0.6589	0.2379	0.5961	0.065*	
C13A	0.4957 (3)	0.1140 (4)	0.63933 (15)	0.0555 (6)	
H13A	0.5492	0.1086	0.6902	0.067*	
C14A	0.3542 (3)	0.0456 (4)	0.62091 (16)	0.0557 (6)	
H14A	0.3104	−0.0069	0.6592	0.067*	
C15A	0.2745 (3)	0.0526 (3)	0.54644 (15)	0.0505 (6)	
H15A	0.1771	0.0044	0.5340	0.061*	
C16A	0.2063 (3)	0.3686 (3)	0.32569 (13)	0.0423 (5)	

### Atomic displacement parameters (Å^2^)

**Table:**

	*U*^11^	*U*^22^	*U*^33^	*U*^12^	*U*^13^	*U*^23^
N1	0.0488 (10)	0.0444 (11)	0.0386 (10)	0.0017 (9)	0.0090 (8)	0.0034 (9)
N2	0.0474 (10)	0.0416 (10)	0.0422 (9)	0.0005 (8)	0.0110 (8)	0.0017 (8)
N3	0.0517 (10)	0.0450 (11)	0.0454 (10)	0.0021 (9)	0.0143 (8)	0.0024 (9)
N4	0.0543 (11)	0.0452 (11)	0.0461 (10)	0.0046 (9)	0.0169 (9)	0.0037 (9)
N5	0.0493 (10)	0.0453 (11)	0.0442 (10)	0.0046 (9)	0.0119 (8)	0.0039 (9)
C1	0.0497 (12)	0.0459 (14)	0.0443 (12)	0.0005 (10)	0.0059 (10)	0.0010 (9)
C2	0.0516 (12)	0.0467 (14)	0.0510 (12)	0.0061 (11)	0.0065 (10)	−0.0033 (11)
C3	0.0506 (12)	0.0575 (15)	0.0458 (12)	0.0035 (11)	0.0125 (9)	−0.0067 (11)
C4	0.0601 (14)	0.0559 (15)	0.0450 (12)	0.0009 (12)	0.0146 (11)	0.0026 (11)
C5	0.0540 (13)	0.0473 (14)	0.0460 (12)	0.0029 (11)	0.0101 (10)	0.0013 (10)
C6	0.0420 (11)	0.0447 (13)	0.0424 (11)	−0.0005 (10)	0.0049 (9)	−0.0017 (9)
C7	0.0407 (10)	0.0424 (12)	0.0446 (11)	−0.0009 (10)	0.0084 (9)	−0.0004 (9)
C8	0.0432 (10)	0.0421 (12)	0.0448 (12)	0.0010 (10)	0.0092 (9)	0.0013 (9)
C9	0.0423 (11)	0.0438 (12)	0.0407 (11)	0.0003 (9)	0.0062 (9)	0.0000 (9)
C10	0.0423 (11)	0.0408 (12)	0.0423 (11)	0.0044 (9)	0.0084 (9)	0.0018 (9)
C11	0.0450 (11)	0.0454 (13)	0.0406 (11)	0.0026 (10)	0.0049 (9)	−0.0001 (9)
C12	0.0465 (11)	0.0457 (13)	0.0499 (13)	−0.0021 (10)	0.0086 (9)	0.0016 (10)
C13	0.0505 (12)	0.0488 (13)	0.0451 (12)	0.0025 (11)	0.0127 (9)	0.0067 (10)
C14	0.0523 (12)	0.0476 (13)	0.0394 (11)	0.0048 (11)	0.0064 (9)	0.0008 (10)
C15	0.0456 (11)	0.0406 (11)	0.0445 (12)	0.0021 (10)	0.0068 (9)	−0.0007 (9)
C16	0.0439 (11)	0.0405 (12)	0.0415 (11)	−0.0031 (9)	0.0081 (9)	0.0016 (9)
N1A	0.0532 (11)	0.0447 (11)	0.0408 (10)	0.0035 (9)	0.0128 (8)	0.0032 (9)
N2A	0.0453 (10)	0.0466 (12)	0.0406 (10)	0.0033 (9)	0.0083 (8)	0.0014 (8)
N3A	0.0518 (11)	0.0496 (11)	0.0437 (10)	0.0050 (9)	0.0114 (8)	0.0004 (9)
N4A	0.0513 (10)	0.0500 (12)	0.0428 (10)	0.0055 (9)	0.0102 (8)	0.0018 (9)
N5A	0.0471 (10)	0.0465 (11)	0.0414 (10)	0.0034 (9)	0.0090 (8)	0.0018 (8)
C1A	0.0479 (12)	0.0451 (12)	0.0440 (11)	0.0001 (10)	0.0087 (9)	0.0042 (10)
C2A	0.0483 (12)	0.0465 (13)	0.0491 (12)	0.0060 (11)	0.0068 (10)	−0.0004 (10)
C3A	0.0410 (11)	0.0537 (14)	0.0448 (12)	0.0037 (10)	0.0086 (9)	−0.0024 (10)
C4A	0.0425 (11)	0.0522 (14)	0.0448 (11)	−0.0022 (10)	0.0066 (9)	0.0007 (11)
C5A	0.0442 (11)	0.0431 (13)	0.0449 (11)	−0.0010 (9)	0.0062 (9)	−0.0001 (10)
C6A	0.0417 (10)	0.0476 (12)	0.0407 (11)	0.0013 (10)	0.0041 (9)	0.0009 (10)
C7A	0.0435 (11)	0.0433 (12)	0.0429 (11)	−0.0009 (10)	0.0058 (9)	−0.0017 (9)
C8A	0.0494 (11)	0.0435 (13)	0.0422 (12)	0.0031 (10)	0.0086 (9)	−0.0002 (9)
C9A	0.0468 (11)	0.0441 (13)	0.0426 (11)	0.0010 (10)	0.0089 (9)	0.0013 (10)
C10A	0.0488 (11)	0.0433 (12)	0.0426 (12)	0.0062 (10)	0.0099 (9)	0.0009 (10)
C11A	0.0539 (13)	0.0469 (13)	0.0482 (13)	0.0008 (11)	0.0110 (10)	0.0030 (10)
C12A	0.0533 (13)	0.0503 (14)	0.0578 (14)	0.0013 (12)	0.0018 (11)	−0.0025 (12)
C13A	0.0658 (15)	0.0560 (15)	0.0433 (12)	0.0136 (13)	0.0033 (11)	−0.0002 (11)
C14A	0.0652 (15)	0.0591 (15)	0.0444 (12)	0.0069 (14)	0.0132 (11)	0.0086 (12)
C15A	0.0514 (12)	0.0529 (14)	0.0484 (13)	0.0023 (12)	0.0114 (10)	0.0032 (11)
C16A	0.0421 (11)	0.0437 (13)	0.0409 (11)	−0.0006 (10)	0.0055 (9)	0.0002 (10)

### Geometric parameters (Å, º)

**Table:**

N1—H1	0.84 (4)	N1A—H1AA	0.84 (4)
N1—C7	1.414 (3)	N1A—C7A	1.399 (4)
N1—C16	1.346 (3)	N1A—C16A	1.351 (3)
N2—N3	1.357 (3)	N2A—N3A	1.356 (3)
N2—C9	1.473 (3)	N2A—C9A	1.473 (3)
N2—C16	1.336 (3)	N2A—C16A	1.338 (3)
N3—N4	1.287 (3)	N3A—N4A	1.285 (3)
N4—N5	1.371 (3)	N4A—N5A	1.367 (3)
N5—C16	1.326 (3)	N5A—C16A	1.326 (4)
C1—H1A	0.9500	C1A—H1AB	0.9500
C1—C2	1.382 (4)	C1A—C2A	1.377 (4)
C1—C6	1.396 (4)	C1A—C6A	1.401 (4)
C2—H2	0.9500	C2A—H2A	0.9500
C2—C3	1.382 (4)	C2A—C3A	1.394 (4)
C3—H3	0.9500	C3A—H3A	0.9500
C3—C4	1.385 (4)	C3A—C4A	1.392 (4)
C4—H4	0.9500	C4A—H4A	0.9500
C4—C5	1.392 (4)	C4A—C5A	1.387 (4)
C5—H5	0.9500	C5A—H5A	0.9500
C5—C6	1.396 (4)	C5A—C6A	1.401 (3)
C6—C7	1.485 (3)	C6A—C7A	1.480 (3)
C7—C8	1.341 (4)	C7A—C8A	1.345 (4)
C8—H8	0.9500	C8A—H8A	0.9500
C8—C9	1.516 (3)	C8A—C9A	1.510 (3)
C9—H9	1.0000	C9A—H9A	1.0000
C9—C10	1.515 (3)	C9A—C10A	1.524 (3)
C10—C11	1.391 (4)	C10A—C11A	1.388 (4)
C10—C15	1.392 (3)	C10A—C15A	1.390 (3)
C11—H11	0.9500	C11A—H11A	0.9500
C11—C12	1.387 (3)	C11A—C12A	1.396 (4)
C12—H12	0.9500	C12A—H12A	0.9500
C12—C13	1.391 (4)	C12A—C13A	1.392 (4)
C13—H13	0.9500	C13A—H13A	0.9500
C13—C14	1.384 (4)	C13A—C14A	1.374 (5)
C14—H14	0.9500	C14A—H14A	0.9500
C14—C15	1.386 (4)	C14A—C15A	1.394 (4)
C15—H15	0.9500	C15A—H15A	0.9500
			
C7—N1—H1	123 (2)	C7A—N1A—H1AA	123 (3)
C16—N1—H1	117 (3)	C16A—N1A—H1AA	118 (3)
C16—N1—C7	118.1 (2)	C16A—N1A—C7A	118.5 (2)
N3—N2—C9	124.36 (19)	N3A—N2A—C9A	124.9 (2)
C16—N2—N3	108.4 (2)	C16A—N2A—N3A	108.1 (2)
C16—N2—C9	126.9 (2)	C16A—N2A—C9A	126.6 (2)
N4—N3—N2	106.25 (19)	N4A—N3A—N2A	106.32 (19)
N3—N4—N5	111.4 (2)	N3A—N4A—N5A	111.6 (2)
C16—N5—N4	104.9 (2)	C16A—N5A—N4A	104.9 (2)
C2—C1—H1A	119.7	C2A—C1A—H1AB	119.7
C2—C1—C6	120.7 (2)	C2A—C1A—C6A	120.5 (2)
C6—C1—H1A	119.7	C6A—C1A—H1AB	119.7
C1—C2—H2	119.7	C1A—C2A—H2A	119.6
C3—C2—C1	120.6 (2)	C1A—C2A—C3A	120.8 (2)
C3—C2—H2	119.7	C3A—C2A—H2A	119.6
C2—C3—H3	120.2	C2A—C3A—H3A	120.5
C2—C3—C4	119.7 (2)	C4A—C3A—C2A	119.0 (2)
C4—C3—H3	120.2	C4A—C3A—H3A	120.5
C3—C4—H4	120.0	C3A—C4A—H4A	119.7
C3—C4—C5	120.0 (3)	C5A—C4A—C3A	120.6 (2)
C5—C4—H4	120.0	C5A—C4A—H4A	119.7
C4—C5—H5	119.7	C4A—C5A—H5A	119.9
C4—C5—C6	120.7 (3)	C4A—C5A—C6A	120.3 (2)
C6—C5—H5	119.7	C6A—C5A—H5A	119.8
C1—C6—C5	118.4 (2)	C1A—C6A—C5A	118.8 (2)
C1—C6—C7	120.6 (2)	C1A—C6A—C7A	119.7 (2)
C5—C6—C7	120.9 (2)	C5A—C6A—C7A	121.5 (2)
N1—C7—C6	115.5 (2)	N1A—C7A—C6A	116.1 (2)
C8—C7—N1	120.8 (2)	C8A—C7A—N1A	120.7 (2)
C8—C7—C6	123.7 (2)	C8A—C7A—C6A	123.2 (2)
C7—C8—H8	117.6	C7A—C8A—H8A	117.5
C7—C8—C9	124.8 (2)	C7A—C8A—C9A	125.0 (2)
C9—C8—H8	117.6	C9A—C8A—H8A	117.5
N2—C9—C8	105.94 (18)	N2A—C9A—C8A	106.3 (2)
N2—C9—H9	107.8	N2A—C9A—H9A	108.7
N2—C9—C10	109.68 (18)	N2A—C9A—C10A	110.8 (2)
C8—C9—H9	107.8	C8A—C9A—H9A	108.7
C10—C9—C8	117.5 (2)	C8A—C9A—C10A	113.40 (19)
C10—C9—H9	107.8	C10A—C9A—H9A	108.7
C11—C10—C9	122.1 (2)	C11A—C10A—C9A	120.5 (2)
C11—C10—C15	119.4 (2)	C11A—C10A—C15A	120.0 (2)
C15—C10—C9	118.4 (2)	C15A—C10A—C9A	119.5 (2)
C10—C11—H11	119.9	C10A—C11A—H11A	120.0
C12—C11—C10	120.2 (2)	C10A—C11A—C12A	120.1 (2)
C12—C11—H11	119.9	C12A—C11A—H11A	120.0
C11—C12—H12	120.0	C11A—C12A—H12A	120.2
C11—C12—C13	120.0 (2)	C13A—C12A—C11A	119.7 (3)
C13—C12—H12	120.0	C13A—C12A—H12A	120.2
C12—C13—H13	120.0	C12A—C13A—H13A	120.0
C14—C13—C12	120.1 (2)	C14A—C13A—C12A	120.1 (2)
C14—C13—H13	120.0	C14A—C13A—H13A	120.0
C13—C14—H14	120.1	C13A—C14A—H14A	119.7
C13—C14—C15	119.9 (2)	C13A—C14A—C15A	120.6 (3)
C15—C14—H14	120.1	C15A—C14A—H14A	119.7
C10—C15—H15	119.8	C10A—C15A—C14A	119.6 (2)
C14—C15—C10	120.5 (2)	C10A—C15A—H15A	120.2
C14—C15—H15	119.8	C14A—C15A—H15A	120.2
N2—C16—N1	121.9 (2)	N2A—C16A—N1A	121.6 (2)
N5—C16—N1	129.1 (2)	N5A—C16A—N1A	129.3 (2)
N5—C16—N2	109.0 (2)	N5A—C16A—N2A	109.1 (2)
			
N1—C7—C8—C9	8.3 (4)	N1A—C7A—C8A—C9A	4.0 (4)
N2—N3—N4—N5	0.1 (3)	N2A—N3A—N4A—N5A	0.3 (3)
N2—C9—C10—C11	−72.2 (3)	N2A—C9A—C10A—C11A	−69.2 (3)
N2—C9—C10—C15	103.6 (2)	N2A—C9A—C10A—C15A	111.3 (3)
N3—N2—C9—C8	−174.5 (2)	N3A—N2A—C9A—C8A	−175.8 (2)
N3—N2—C9—C10	−46.7 (3)	N3A—N2A—C9A—C10A	−52.2 (3)
N3—N2—C16—N1	−179.7 (2)	N3A—N2A—C16A—N1A	−178.4 (2)
N3—N2—C16—N5	1.4 (3)	N3A—N2A—C16A—N5A	0.6 (3)
N3—N4—N5—C16	0.7 (3)	N3A—N4A—N5A—C16A	0.0 (3)
N4—N5—C16—N1	179.9 (2)	N4A—N5A—C16A—N1A	178.5 (2)
N4—N5—C16—N2	−1.3 (3)	N4A—N5A—C16A—N2A	−0.4 (3)
C1—C2—C3—C4	0.3 (4)	C1A—C2A—C3A—C4A	0.2 (4)
C1—C6—C7—N1	151.9 (2)	C1A—C6A—C7A—N1A	−149.0 (2)
C1—C6—C7—C8	−30.0 (4)	C1A—C6A—C7A—C8A	29.2 (4)
C2—C1—C6—C5	−0.3 (4)	C2A—C1A—C6A—C5A	−0.4 (4)
C2—C1—C6—C7	176.9 (2)	C2A—C1A—C6A—C7A	178.3 (2)
C2—C3—C4—C5	−0.6 (4)	C2A—C3A—C4A—C5A	−0.5 (4)
C3—C4—C5—C6	0.5 (4)	C3A—C4A—C5A—C6A	0.3 (4)
C4—C5—C6—C1	−0.1 (4)	C4A—C5A—C6A—C1A	0.1 (4)
C4—C5—C6—C7	−177.2 (2)	C4A—C5A—C6A—C7A	−178.5 (2)
C5—C6—C7—N1	−31.0 (3)	C5A—C6A—C7A—N1A	29.6 (3)
C5—C6—C7—C8	147.1 (3)	C5A—C6A—C7A—C8A	−152.2 (2)
C6—C1—C2—C3	0.2 (4)	C6A—C1A—C2A—C3A	0.2 (4)
C6—C7—C8—C9	−169.7 (2)	C6A—C7A—C8A—C9A	−174.1 (2)
C7—N1—C16—N2	−2.1 (3)	C7A—N1A—C16A—N2A	−4.6 (3)
C7—N1—C16—N5	176.6 (2)	C7A—N1A—C16A—N5A	176.6 (2)
C7—C8—C9—N2	−14.0 (3)	C7A—C8A—C9A—N2A	−10.7 (3)
C7—C8—C9—C10	−137.0 (3)	C7A—C8A—C9A—C10A	−132.7 (3)
C8—C9—C10—C11	48.8 (3)	C8A—C9A—C10A—C11A	50.3 (3)
C8—C9—C10—C15	−135.3 (2)	C8A—C9A—C10A—C15A	−129.2 (2)
C9—N2—N3—N4	−174.4 (2)	C9A—N2A—N3A—N4A	−174.9 (2)
C9—N2—C16—N1	−6.4 (4)	C9A—N2A—C16A—N1A	−4.2 (4)
C9—N2—C16—N5	174.7 (2)	C9A—N2A—C16A—N5A	174.8 (2)
C9—C10—C11—C12	175.2 (2)	C9A—C10A—C11A—C12A	−179.6 (2)
C9—C10—C15—C14	−176.1 (2)	C9A—C10A—C15A—C14A	−179.9 (3)
C10—C11—C12—C13	1.2 (4)	C10A—C11A—C12A—C13A	−0.3 (4)
C11—C10—C15—C14	−0.2 (4)	C11A—C10A—C15A—C14A	0.6 (4)
C11—C12—C13—C14	−1.0 (4)	C11A—C12A—C13A—C14A	0.4 (4)
C12—C13—C14—C15	0.3 (4)	C12A—C13A—C14A—C15A	0.0 (5)
C13—C14—C15—C10	0.3 (4)	C13A—C14A—C15A—C10A	−0.5 (4)
C15—C10—C11—C12	−0.6 (3)	C15A—C10A—C11A—C12A	−0.2 (4)
C16—N1—C7—C6	179.1 (2)	C16A—N1A—C7A—C6A	−177.2 (2)
C16—N1—C7—C8	1.0 (3)	C16A—N1A—C7A—C8A	4.5 (3)
C16—N2—N3—N4	−0.9 (3)	C16A—N2A—N3A—N4A	−0.5 (3)
C16—N2—C9—C8	13.2 (3)	C16A—N2A—C9A—C8A	10.9 (3)
C16—N2—C9—C10	141.0 (2)	C16A—N2A—C9A—C10A	134.5 (2)

### Hydrogen-bond geometry (Å, º)

**Table:**

*D*—H···*A*	*D*—H	H···*A*	*D*···*A*	*D*—H···*A*
N1—H1···N5*A*	0.84 (4)	2.16 (4)	2.952 (3)	157 (3)
N1*A*—H1*AA*···N5	0.84 (4)	2.10 (4)	2.912 (3)	163 (4)
