# Supplementary figures and images for: Crystal structure of (S)-5,7-diphenyl-4,7-di­hydro­tetra­zolo[1,5-a]pyrimidine
Source: Acta Crystallogr E Crystallogr Commun. 2015 Mar 4;71(Pt 4):o220–1. doi: 10.1107/S2056989015002996 (PMC4438826; doi:10.1107/S2056989015002996)

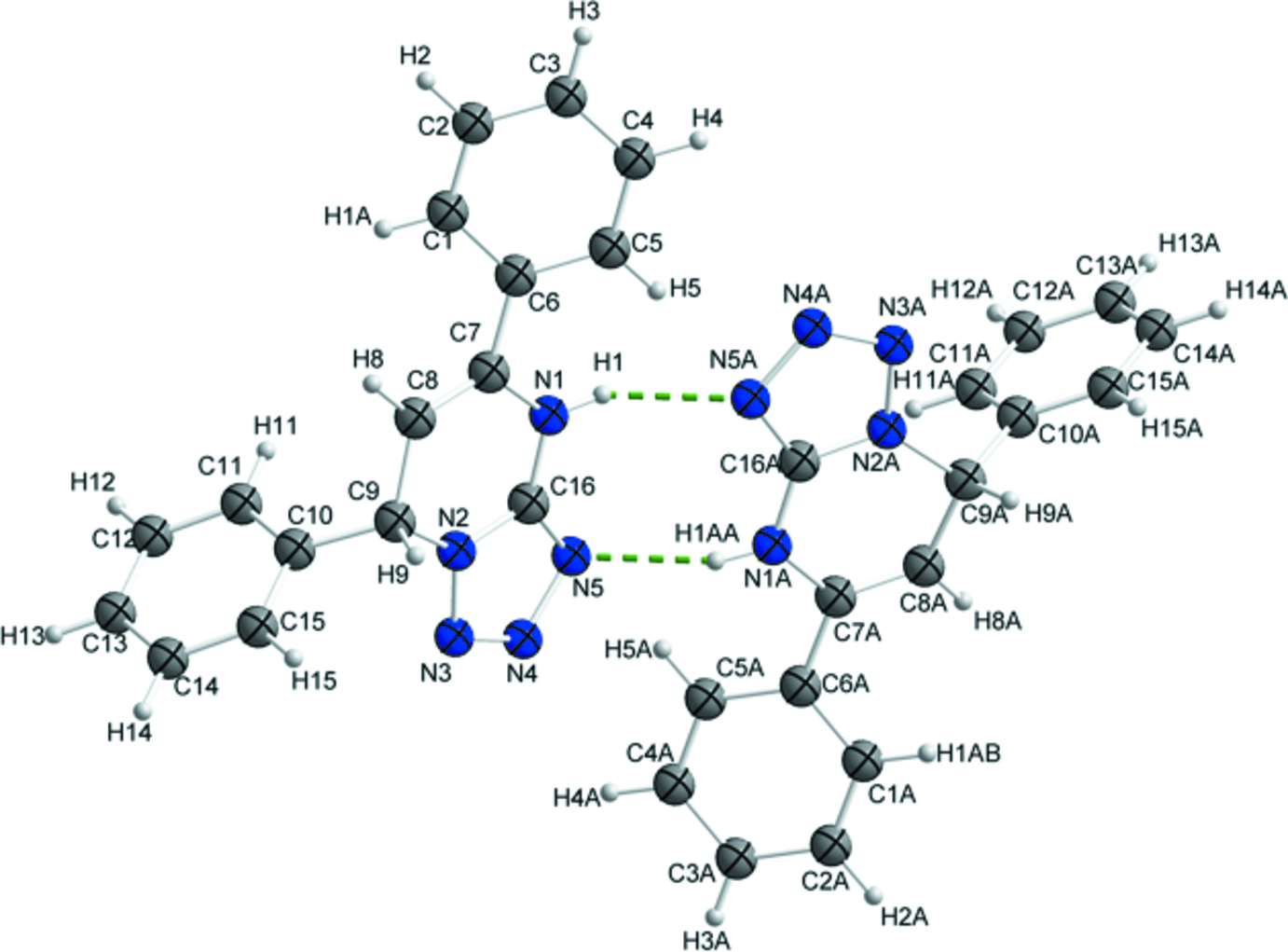

Supplement: Supplementary file 5 [file e-71-0o220-fig1.tif]
